# 32460 The Bench Tutorials Program: An Essential Educational Pivot in response to COVID-19

**DOI:** 10.1017/cts.2021.419

**Published:** 2021-03-30

**Authors:** Chantele Singleton, Sharon A. Croisant, Lance Hallberg, John Prochaska, Krista Bohn, Michelle Puig, Cornelis Elferink

**Affiliations:** 1 The University of Texas Medical Branch; 2 Galveston Independent School District

## Abstract

ABSTRACT IMPACT: The Bench Tutorials Program is an independent study course in biomedical research in which high school students are paired with graduate and post-doctoral students during the academic year. The purpose is to enhance the rigor of high school science education and build the pipeline of tomorrow’s researchers. OBJECTIVES/GOALS: The Bench Tutorials Program: ο Proficiency in research design, implementation, and presentation; ο Acquisition of hands-on laboratory skills; ο Increase in scientific literacy; ο Increase in analytical skills and critical thinking; ο Career in science; ο Build the pipeline of tomorrow’s biomedical researchers METHODS/STUDY POPULATION: High School seniors are paired with graduate and postdoc mentors through a matching process. Students spend approximately four hours/week in supervised instruction and research from a participating laboratory in addition to classroom experience at their High School. Mentors design research projects relating to the larger research framework of their laboratories. In light of COVID-19, approaches have been adjusted to maintain the program safely through a hybrid method of using the high school lab for hands-on learning and through the use of Go-Pros ’s to enable our mentors to video and narrate as they conduct experiments in their own labs to teach their mentees scientific methods and processes. RESULTS/ANTICIPATED RESULTS: Since inception, more than 400 students and mentors have participated in the Bench Tutorial’s program. This year we found a way to continue the program under COVID-19 restraints without putting anyone in harms way. Go-Pros have been essential for our program to maintain continuity for high school students who receive academic credit for this course. This program is also one of few in which our graduate students have the opportunity to serve as mentors in the scientific setting. Using Go-Pro’s will also enable us to provide teaching videos online for other academic institutions, so even in the absence of COVID-19 in the future, the continued use of these devices will still be of great value. DISCUSSION/SIGNIFICANCE OF FINDINGS: High school students are afforded the ability to work on cutting edge research projects alongside graduate students and postdocs, who are afforded the chance to mentor and teach. Due to the COVID-19 pandemic, we have successfully adjusted our methods for teaching through the use of Go-Pro technology.

